# The prevalence of patients with rheumatic diseases and its periodontal condition: data from a population-based study in the Lisbon Metropolitan Area

**DOI:** 10.1080/07853890.2021.1897396

**Published:** 2021-09-28

**Authors:** João Botelho, Vanessa Machado, Luís Proença, Ricardo Alves, Maria Alzira Cavacas, José João Mendes

**Affiliations:** a Periodontology Department, Clinical Research Unit (CRU), Centro de Investigacão Interdisciplinar Egas Moniz (CiiEM), Egas Moniz Cooperativa de Ensino Superior, Caparica, Portugal; b Clinical Research Unit (CRU), Centro de Investigacão Interdisciplinar Egas Moniz (CiiEM), Egas Moniz Cooperativa de Ensino Superior, Caparica, Portugal; c Quantitative Methods for Health Research (MQIS), Centro de Investigacão Interdisciplinar Egas Moniz (CiiEM), Egas Moniz Cooperativa de Ensino Superior, Caparica, Portugal

## Abstract

**Introduction:**

Periodontitis is a major condition associated with rheumatic diseases (RDs) [1] and some studies have clarified the effect of the oral microbiome in RDs [2,3]. However, due to the lack of information this observational study aimed to describe the periodontal status of RDs in a sample of patients from a population-based epidemiologic survey carried out in the southern Lisbon Metropolitan Area.

**Materials and Methods:**

From December 2018 to April 2019, a total of 1064 patients, from public health centres of Almada-Seixal Group of Centres, were randomly enrolled in the study. RDs were assessed through a medical history and medication questionnaire. Periodontitis and Gingivitis were circumferentially evaluated according to the 2018 World Case Definitions [4,5] by two calibrated examiners (J.B. and V.M). This study was approved by the ARSLVT Ethics Committee (3525 & 8696/CES/2018).

**Results:**

Overall, the prevalence of rheumatic conditions was 2.8% (95% CI: 1.8–3.8%) (*n* = 30). Individual RD prevalence distribution in the study group were as follows: rheumatoid arthritis (RA) 23.3% (*n* = 7), fibromyalgia (FM) 36.7% (*n* = 11), systemic lupus erythematosus (SLE) 10.0% (*n* = 3), arthritis (ART) 13.3% (*n* = 4), gout 3.3% (*n* = 1), systemic scleroderma 3.3% (*n* = 1), FM + osteoarthritis (OA) 3.3% (*n* = 1), FM + SLE 3.3% (*n* = 1), FME + OA + ART 3.3% (*n* = 1). The prevalence of periodontitis among RD patients was 60% (*n* = 18), with 13.3% (*n* = 4), 16.7% (*n* = 5) and 26.7% (*n* = 8) of mild, moderate and severe stages, respectively. Gingivitis cases were residual, 3.3% (*n* = 1). The average missing teeth were 10.7 (±6.8) and the mean percentage of probing depth ≥4 mm was 5.7% (±10.9%).

**Discussion and conclusions:**

Despite the low incidence of RDs, these results reveal a considerable high prevalence of periodontitis and gingivitis among those patients. Also, the average number of missing teeth is worrisome. These findings unveil a very disturbing high burden of periodontitis in this sample of Portuguese rheumatic patients and roots basis for future public health measures implementation.
